# Beyond explainability: toward trustworthy governance of generative AI in digital public health surveillance

**DOI:** 10.3389/fpubh.2026.1901894

**Published:** 2026-07-13

**Authors:** R. Sona, S. R. Sanjith, M. Iswarya, M. Bhuvaneswari

**Affiliations:** 1 LEAD College of Management, Palakkad, India; 2 Chinmaya Vishwavidyapeeth Deemed to be University, Kochi, India; 3 Hindustan College of Engineering and Technology, Coimbatore, India; 4 Karpagam Academy of Higher Education (Deemed to be University), Coimbatore, India

**Keywords:** AI governance, digital health, explainable AI, generative artificial intelligence, health policy, public health surveillance, trustworthy AI

## 1 Introduction

Artificial intelligence has become an indispensable component of modern public health surveillance. During the COVID-19 pandemic, AI-supported systems assisted governments and health agencies in forecasting outbreaks, identifying vulnerable populations, allocating healthcare resources, and supporting evidence-based policymaking (1). These applications demonstrated the transformative potential of data-driven technologies in strengthening public health preparedness and response. Recent advances in generative artificial intelligence have expanded these capabilities considerably. Unlike traditional predictive models that primarily classify, forecast, or detect patterns, generative systems can synthesize information, generate reports, create synthetic datasets, and produce epidemiological intelligence from large volumes of unstructured data (2). Public health organizations are increasingly exploring these technologies for outbreak monitoring, health communication, decision support, and knowledge management. However, the rapid integration of generative AI introduces challenges that extend far beyond conventional concerns regarding accuracy and transparency. In this article, explainability refers to the ability of AI systems to provide understandable reasons for their outputs and recommendations. Governance refers to the institutional, ethical, legal, and operational mechanisms that guide the development, deployment, oversight, and accountability of AI systems. Explainable AI has emerged as a dominant strategy for improving trust in AI systems by making model decisions more understandable. While explainability remains valuable, it is fundamentally insufficient to address broader issues related to accountability, fairness, institutional legitimacy, public trust, and societal impact (3). This opinion article advances a strong and timely argument: governance, not explainability, will determine the long-term success of generative AI in digital public health surveillance. As generative AI systems become increasingly influential in shaping health information and public decision-making, governance frameworks must evolve rapidly to ensure responsible, equitable, and trustworthy deployment. The public health community cannot afford to treat AI governance as a secondary concern or technical afterthought. The perspectives presented in this opinion article are informed by the authors' academic engagement in digital public health, health policy, and the emerging applications of artificial intelligence in healthcare and public health systems. Our arguments draw upon contemporary evidence and ongoing scholarly discussions concerning trustworthy and responsible AI governance. Despite growing attention to explainable AI, there remains a lack of comprehensive governance frameworks tailored specifically for generative AI in digital public health surveillance.

## 2 Why explainability alone is not enough

Explainability has become a central pillar of responsible AI development (4). Techniques such as feature importance analysis, attention visualization, and *post-hoc* interpretation methods seek to make AI outputs more transparent. In healthcare settings, explainability can improve clinician confidence and facilitate understanding of algorithmic recommendations. Despite these benefits, explainability has critical and often overlooked limitations that become particularly dangerous in public health contexts. Transparent systems can still produce biased outcomes, reinforce existing inequalities, and generate harmful recommendations (5). Knowing how a model reached a decision does not necessarily justify the fairness or appropriateness of that decision. An algorithm might have a perfectly clear explanation for why it prioritized one population over another, yet that decision could still reflect historical biases embedded in training data. Furthermore, explanations may create a false sense of confidence, encouraging users to trust outputs without critically evaluating their validity. This phenomenon is particularly concerning in public health, where decisions affect entire populations and mistakes can have catastrophic consequences. Clinicians and public health officials may interpret technical explanations as validation of correctness, even when underlying data or assumptions are flawed. Generative AI introduces additional and more severe concerns. Large language models can generate plausible but inaccurate information, commonly referred to as hallucinations. In public health contexts, such outputs could influence risk communication, resource allocation, and policy responses (6). A generative AI system might confidently produce an epidemiological report with fabricated case numbers, distorted transmission patterns, or incorrect treatment recommendations. Explainability alone cannot prevent misinformation, resolve accountability disputes, or address population-level harms (7). Consider a scenario where a generative AI system is deployed to synthesize outbreak reports from multiple countries. The system might generate a coherent and well-explained summary, yet contain critical errors that misdirect public health responses. Knowing which words, the model attended to or which features influenced its output does nothing to prevent the spread of dangerous misinformation. An explanation of a wrong answer is still a wrong answer. Consequently, the primary challenge is no longer understanding how AI systems function but determining how they should be governed within complex public health environments. The question is not whether we can explain AI decisions, but whether we can ensure those decisions are safe, equitable, and accountable.

## 3 From explainability to trustworthy AI governance

Trustworthy AI extends beyond transparency and incorporates principles such as fairness, accountability, privacy, safety, human oversight, and public engagement (8). Governance provides the institutional mechanisms necessary to operationalize these principles. Without governance, principles remain abstract ideals with no practical mechanism for implementation or enforcement. Effective governance frameworks must define responsibilities across the entire AI lifecycle, including system design, deployment, monitoring, auditing, and evaluation. Such frameworks must establish clear mechanisms for identifying bias, addressing harms, protecting privacy, and ensuring compliance with ethical and legal standards. Governance must be proactive rather than reactive, anticipating risks before deployment rather than responding to crises after harms occur. Governance is particularly critical in public health because decisions frequently affect entire populations rather than individual users. Public health institutions must therefore balance innovation with equity, accountability, and social responsibility. The stakes are fundamentally different from commercial AI applications (9). A biased recommendation system might inconvenience a consumer, but a biased public health AI system might deny care to vulnerable populations or misallocate scarce resources during an outbreak. Without robust governance, the benefits of generative AI may be undermined by misinformation, algorithmic discrimination, and declining public trust. Public trust in health institutions is already fragile in many contexts, and the introduction of opaque or harmful AI systems could erode this trust further. Once lost, public trust is extraordinarily difficult to rebuild, and its absence can undermine entire public health programs. The public health community must recognize that AI governance is not a technical problem to be solved by engineers, but a public health responsibility that requires multidisciplinary expertise, institutional commitment, and sustained investment.

## 4 Opportunities and risks of generative AI in public health surveillance

Generative AI offers substantial and transformative opportunities for improving public health surveillance (10). These technologies can transform unstructured outbreak reports into structured knowledge systems, automate information synthesis, support multilingual communication, and generate privacy-preserving synthetic datasets for research and planning purposes (11). The potential benefits are enormous. GenAI systems could analyze millions of social media posts, news articles, and clinical reports to detect emerging disease patterns weeks before traditional surveillance systems. They could generate real-time multilingual health communication for diverse populations during outbreaks. They could create synthetic patient data that preserves statistical properties while protecting individual privacy, enabling research that would otherwise be impossible due to privacy constraints. At the same time, significant and potentially catastrophic risks remain. Hallucinated outputs may spread inaccurate health information at unprecedented speed and scale. Biased training data can amplify existing disparities among marginalized populations, systematically disadvantaging those already most vulnerable. Malicious actors may exploit generative systems to create persuasive health misinformation that is difficult to distinguish from authentic content. In addition, concerns regarding privacy, cybersecurity, environmental sustainability, and regulatory oversight remain largely unresolved. The combination of high influence and high uncertainty makes public health one of the most sensitive domains for generative AI deployment. Public health decisions affect millions of lives, influence economic activity, shape political discourse, and determine resource allocation during crises. Generative AI systems deployed in these contexts carry risks that extend far beyond individual harm to population-level consequences. Furthermore, the speed of GenAI deployment in public health is outpacing the development of appropriate governance frameworks. Technology vendors are eager to deploy systems, public health organizations are under pressure to adopt innovative solutions, and regulatory bodies are struggling to keep pace (12). This imbalance creates a dangerous situation where powerful technologies are being deployed without adequate safeguards. Consequently, governance frameworks must evolve at the same pace as technological innovation, not years behind it. The public health community cannot afford to repeat the mistakes of earlier technology deployments where harms emerged only after widespread adoption.

## 5 The TRUST-GPH framework for generative AI in public health intelligence

To address these challenges, this article proposes a comprehensive six-pillar governance framework for generative AI in digital public health surveillance. This framework is designed to be actionable, context-adaptable, and readily implementable by public health institutions. To facilitate policy adoption and scholarly discussion, we propose the TRUST-GPH Framework (Trustworthy Use of Generative AI for Public Health Intelligence). The term “TRUST” is used as an overarching conceptual anchor to represent trustworthy and responsible AI governance in public health, rather than a strict letter-by-letter acronym mapping. The framework comprises six interdependent pillars that collectively promote the responsible, equitable, and trustworthy deployment of generative AI within digital public health surveillance systems (Table 1). The pillars are non-hierarchical and should not be interpreted as a sequence of priorities. Rather, they function as mutually reinforcing governance mechanisms in which weaknesses in one pillar can undermine the effectiveness of the others.

**Table 1:** The TRUST-GPH framework: governance pillars, objectives, actions, and expected outcomes.

Pillar	Objective	Implementation actions	Expected outcomes
Equity-Centered design	Minimize bias and promote health equity	Use representative datasets, conduct bias audits, include marginalized populations	Fair and inclusive AI systems
Accountability and liability	Define responsibilities and mechanisms for redress	Assign roles to developers, deployers and regulators; establish reporting systems	Increased accountability and trust
Human-in-the-loop oversight	Maintain expert supervision of AI decisions	Require human review of critical outputs and override mechanisms	Safer and context-sensitive decisions
Continuous auditing and monitoring	Ensure ongoing evaluation of AI performance	Independent audits, monitoring dashboards, periodic reassessments	Reliable and adaptive systems
Transparency and public engagement	Promote openness and societal trust	Public communication, stakeholder consultations, disclosure practices	Improved public trust and legitimacy
Regulatory and ethical alignment	Ensure legal and ethical compliance	Align systems with privacy, autonomy, beneficence and justice principles	Responsible and sustainable AI governance

*Equity-centered design* requires that AI systems be developed using diverse and representative datasets to minimize bias and promote health equity across populations. This means actively including data from marginalized communities, auditing training data for representation gaps, and prioritizing equity metrics alongside accuracy metrics during development. Equity cannot be an afterthought; it must be embedded in the design process from the beginning. This pillar also requires routine equity impact assessments and continuous monitoring of algorithmic performance across different demographic groups. *Accountability and liability* demand clear responsibilities assigned to developers, deployers, regulators, and public health organizations, with mechanisms for redress when AI systems cause harm. A fundamental governance question concerns who bears responsibility when an AI system causes harm. Current ambiguity in this area creates a dangerous accountability vacuum that allows harmful systems to operate without consequence. Clear accountability pathways should include reporting mechanisms, remediation procedures, and legal frameworks specifying responsibility for AI-related harms. *Human-in-the-loop oversight* ensures that critical public health decisions remain subject to expert human judgment, with AI augmenting rather than replacing professional decision-making. No AI system should possess unchecked authority to make decisions affecting population health. Human experts must remain in the decision loop, with the authority to override AI recommendations when necessary. Human oversight should be supported by adequate workforce training and clearly defined escalation procedures for high-risk situations. *Continuous auditing and monitoring calls* for independent audits evaluating system performance, fairness, safety, and reliability throughout the AI lifecycle. Systems must be audited before deployment, during operation, and after modifications. Audits must be conducted by independent parties with no conflict of interest, and results should be transparently disclosed to relevant stakeholders where appropriate. Auditing frameworks should include predefined performance indicators and trigger mechanisms for system modification or suspension when risks are identified. *Transparency and public engagement* require public health organizations to communicate openly about AI use, limitations, and governance practices while involving stakeholders in decision-making processes. Communities have a right to know when AI systems are being used to make decisions affecting their health. Public engagement must be meaningful, not tokenistic, with genuine opportunities for community input. Public communication should explain both the benefits and limitations of AI systems in language that is accessible to non-technical audiences. *Regulatory and ethical alignment* mandates that AI deployment comply with emerging regulatory frameworks and established ethical principles, including privacy, autonomy, beneficence, and justice. Public health AI must satisfy not only technical requirements but also ethical obligations to populations. Regulatory approaches should remain adaptive so that governance mechanisms can evolve alongside rapidly advancing AI technologies. The six pillars are highly interconnected. Equity-centered design influences the fairness of system outputs and therefore affects public trust and accountability. Human oversight relies on transparent system documentation and continuous monitoring to identify errors and biases. Regulatory alignment provides the institutional foundation upon which accountability mechanisms and public engagement processes can operate. Consequently, effective governance requires simultaneous attention to all six pillars rather than isolated implementation of individual components. Together, these six pillars provide a robust foundation for trustworthy and socially responsible AI governance. They are not abstract ideals but concrete requirements that can be operationalized, measured, and enforced. The TRUST-GPH Framework is intended to serve as an implementation-oriented governance framework that guides the design, deployment, evaluation, and oversight of generative AI applications across diverse public health settings. The acronym TRUST-GPH is intended to signify the overarching goal of fostering trustworthy generative AI for public health intelligence rather than representing a direct one-to-one mapping with the six governance pillars.

## 6 Discussion and conclusion

Governance of generative AI must be recognized as a core public health responsibility rather than merely a technical concern. Public health institutions have long been responsible for protecting populations from emerging risks, from infectious diseases to environmental hazards to food safety. AI-related harms should be viewed within the same framework. Just as public health agencies regulate vaccines, pharmaceuticals, and medical devices, they must also regulate AI systems deployed in public health contexts. Governments, regulators, healthcare organizations, researchers, and technology developers must collaborate to establish governance structures that prioritize public benefit over technological enthusiasm. Too often, AI deployment is driven by vendor interests, institutional prestige, or the fear of falling behind, rather than by evidence of public health benefit. Public education initiatives must improve AI literacy and help individuals critically evaluate AI-generated health information. Workforce training programs must equip public health professionals with the knowledge required to evaluate and supervise AI systems effectively. International collaboration will also be essential. Since digital health technologies frequently operate across borders, governance approaches must encourage cooperation while remaining sensitive to local contexts and health priorities. Disease outbreaks do not respect national boundaries, and neither should AI governance. International standards, shared auditing mechanisms, and cross-border information sharing will be essential for effective governance. Operationalizing the TRUST-GPH Framework requires action at institutional, national, and international levels. At the institutional level, public health organizations should establish multidisciplinary AI governance committees comprising public health experts, clinicians, ethicists, legal scholars, data scientists, and community representatives. At the national level, governments should develop regulatory standards for algorithmic auditing, transparency reporting, and accountability mechanisms. At the international level, organizations should collaborate to establish interoperable governance standards, shared auditing frameworks, and cross-border information-sharing mechanisms that support coordinated responses to global public health emergencies. Implementation should occur through a phased approach beginning with pilot projects, followed by independent evaluation and progressive scaling of successful governance practices. The public health community must also courageously acknowledge that not all AI applications should be deployed, regardless of technical performance. Some use cases may present risks that outweigh benefits, and public health institutions must have the authority and willingness to reject harmful applications. Caution is not innovation-stifling; it is responsible stewardship. Digital public health surveillance is entering a new era shaped by generative artificial intelligence. While explainability remains an important component of responsible AI, it is no longer sufficient to address the broader ethical, social, and institutional challenges associated with generative systems. The field must move beyond the false promise that transparency alone will solve problems of accountability, fairness, and public trust. The future success of generative AI in public health will depend not only on technological performance but also on the strength of governance mechanisms that guide its development and deployment. Governance frameworks that promote accountability, equity, transparency, human oversight, and public participation are essential for ensuring that AI serves the public good. Ultimately, the defining challenge of the generative AI era is not whether these technologies can produce intelligent outputs, but whether societies can establish governance systems capable of ensuring that those outputs remain trustworthy, equitable, and aligned with public health values. This is a challenge that the public health community cannot defer to others. We must lead this effort, drawing on our expertise in population health, our experience with risk management, and our commitment to health equity. The time for action is now. Generative AI systems are already being deployed in public health contexts worldwide (13). Every day of delay in establishing robust governance frameworks increases the risk of harm and erodes public trust. The public health community must rise to this challenge with the same urgency, collaboration, and commitment to population health that it has demonstrated in previous public health emergencies. Governance is not optional. It is the foundation upon which trustworthy AI in public health must be built. Future empirical research should evaluate the feasibility and effectiveness of the TRUST-GPH Framework across different health systems and governance contexts.
